# The Safety, Tolerability, and Preliminary Efficacy of a Gemcitabine-releasing Intravesical System (TAR-200) in American Urological Association–defined Intermediate-risk Non–muscle-invasive Bladder Cancer Patients: A Phase 1b Study

**DOI:** 10.1016/j.euros.2024.01.013

**Published:** 2024-02-16

**Authors:** F. Johannes P. van Valenberg, Antoine G. van der Heijden, Christopher J. Cutie, Sumeet Bhanvadia, Kirk A. Keegan, Shalaka Hampras, Hussein Sweiti, John C. Maffeo, Shu Jin, Albert Chau, Donald L. Reynolds, Crysti Iarossi, April Kelley, Xiang Li, Katharine A. Stromberg, J.P. Michiel Sedelaar, Jessica J.O. Steenbruggen, Diederik M. Somford, J. Alfred Witjes

**Affiliations:** a Department of Urology, Radboudumc, Nijmegen, The Netherlands; b Department of Urology, Canisius Wilhelmina Hospital, Nijmegen, The Netherlands; c Janssen Research & Development, Lexington, MA, USA; d Department of Urology, Vanderbilt University, Nashville, TN, USA; e Janssen Research & Development, Raritan, NJ, USA; f Janssen Research & Development, Spring House, PA, USA; g Datacision Limited, London, UK

**Keywords:** Gemcitabine, Intravesical drug delivery system, Intravesical therapy, Non–muscle-invasive bladder cancer, Prolonged exposure, TAR-200

## Abstract

**Background and objective:**

Patients with intermediate-risk non–muscle-invasive bladder cancer (IR NMIBC) have a high risk of recurrence and need effective therapies to reduce the risk of disease recurrence or progression. This phase 1b study (NCT02720367) assessed the safety and tolerability of TAR-200, an intravesical drug delivery system, in participants with IR NMIBC.

**Methods:**

Participants with recurrent IR NMIBC were eligible. Participants received either two 7-d or two 21-d TAR-200 dosing cycles over a 4–6-wk period in a marker lesion/ablation design. TAR-200 was placed in the window between the cystoscopy showing recurrent papillary disease and the subsequent complete transurethral resection of the bladder tumour. The primary endpoint was TAR-200 safety. The secondary endpoints included TAR-200 tolerability, pharmacokinetics, and preliminary efficacy.

**Key findings and limitations:**

Twelve participants received TAR-200 treatment. No TAR-200–related serious or grade ≥ 3 treatment-emergent adverse events (TEAEs) occurred. Nine participants had grade ≤ 2 TAR-200–related TEAEs, with urgency, dysuria, and haematuria being most common. Two participants refused a second dosing cycle due to urinary urgency and frequency. Insertion and removal of TAR-200 was successful in all cases. Plasma gemcitabine concentrations remained below the lower limit of detection. Five participants (42%) had complete response (CR): four had pathological CR and one had CR based on visual assessment.

**Conclusions and clinical implications:**

TAR-200 appears to be safe and well tolerated, with encouraging preliminary efficacy in participants with IR NMIBC. This study lays the groundwork for the multiple phase 2 and 3 global studies that are currently on-going for TAR-200.

**Patient summary:**

In this study, researchers evaluated the safety of the novel drug delivery system TAR-200 in participants with intermediate-risk non-muscle-invasive bladder cancer. They concluded that TAR-200 was safe and well tolerated with promising antitumour activity.

## Take Home Message

In this phase 1b study, TAR-200, a novel drug delivery system, was safe and well tolerated in participants with intermediate-risk non–muscle-invasive bladder cancer. TAR-200 demonstrated promising antitumour activity via direct papillary tumour ablation after two consecutive TAR-200 placements prior to transurethral resection of the bladder tumour.

## 1 Introduction

Bladder cancer represents a significant public health burden, ranking as the sixth most frequently diagnosed cancer and 11th most deadly cancer in the USA, with worldwide incidence rates being highest in Southern and Western Europe [1], [2]. Approximately 75% of incident cases are non–muscle-invasive bladder cancer (NMIBC) at initial diagnosis, often requiring intensive treatment regimens to reduce the risk of recurrence or progression to muscle-invasive bladder cancer (MIBC) [3], [4]. European Association of Urology (EAU) guidelines classify NMIBC according to risk groupings based on tumour characteristics, clinical features, and risk factors (low risk [LR], intermediate risk [IR], high risk [HR], or very HR) [4]. American Urological Association (AUA) guidelines take a similar approach with classifications of LR, IR, and HR [5]. These classifications provide the basis for recommendations on treatment, management, and follow-up [3], [4], [5].

The standard of care for IR NMIBC is transurethral resection of the bladder tumour (TURBT) or bladder biopsy followed by intravesical chemo- or immunotherapeutic instillations and serial surveillance with cystoscopy [4], [6], [7]. Despite this, within 5 yr of initial diagnosis, ∼67% of patients with IR NMIBC will experience recurrent disease [8]. The high prevalence rates and health care-related costs of NMIBC are due to the frequency of recurrences and a lower risk of NMIBC-specific mortality, leading to prolonged follow-up with frequent surveillance procedures [9], [10]. Therefore, there is a need for more effective strategies to decrease treatment burden on patients, improve efficacy, and reduce recurrence.

The current approach of frequent intravesical instillations is limited by the inability to uniformly expose the bladder to intravesical agents for longer than 1–2 h due to limited dwell times and subsequent patient voiding. Given that the mean cell-cycle duration of tumours may be 48 h, classic, episodic delivery of short-term intravesical instillations falls well short of optimal exposure to adequately impair growing cancer cells [11]. Most commonly, mitomycin C, epirubicin, or gemcitabine has been utilised for intravesical chemotherapy instillations [6]. Gemcitabine has proven efficacy in NMIBC [12]. It is utilised as an agent for intravesical delivery due to its unique pharmacological properties and molecular mass that may enable better penetration of bladder mucosa while also minimising significant systemic absorption, as well as its improved tolerability, global availability, and reduced treatment costs [12], [13], [14]. Additionally, the risk of systemic toxicity is minimised with local delivery, as any systemic gemcitabine is metabolised readily by renal excretion [12], [15].

**Fig. 1:**
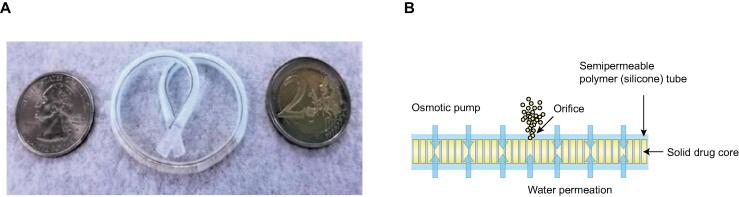
(A) TAR-200 consists of a small, flexible silicone delivery system filled with gemcitabine and osmotic mini-tablets. This figure was published in the study of Daneshmand et al [14], copyright Elsevier (2022). (B) Mechanism of action of the TAR-200 osmotic pump: the solid-state drug and salts located in the semipermeable silicone tube create an osmotic pump that drives the aqueous component of urine through 225 mg of gemcitabine mini-tablets. The dissolved gemcitabine is driven out through a central orifice in the TAR-200, releasing drug in a sustained, controlled, and low-dose manner for over > 7 d.

TAR-200 is a novel intravesical drug delivery system that provides continuous, controlled, low-dose local delivery of gemcitabine into the bladder (Fig. 1) [14], [16]. With this drug delivery system, gemcitabine disseminates into intravesical urine and is transported into the deep tissue layers of the bladder with sustained local dosing [14], [16]. Preclinical studies revealed that continuous low-dose intravesical gemcitabine infusion inhibits MIBC in a concentration-dependent manner [16], [17]. An important benefit is that TAR-200 delivers a substantially lower total dose of gemcitabine than standard intravesical gemcitabine instillations, resulting in potentially reduced bladder and systemic toxicity [18], [19]. In previous studies, TAR-200 was shown to be safe and well tolerated, and demonstrated preliminary efficacy in participants with MIBC [14], [16]. Pharmacokinetic (PK) data from the phase 1 TAR-200-101 study demonstrated that gemcitabine and its metabolite, 2′,2′-difluorodeoxyuridine (dFdU), were detected appropriately in urine, while gemcitabine was not detectable in plasma, and measurable dFdU plasma levels were very low [14].

The aim of this phase 1b study in participants with IR NMIBC was to evaluate the safety, tolerability, and preliminary direct antitumour effect of the continuous release of intravesical gemcitabine, as delivered by TAR-200, in a marker lesion/ablation study design.

## 2 Patients and methods

### 2.1 Study design

**Fig. 2:**
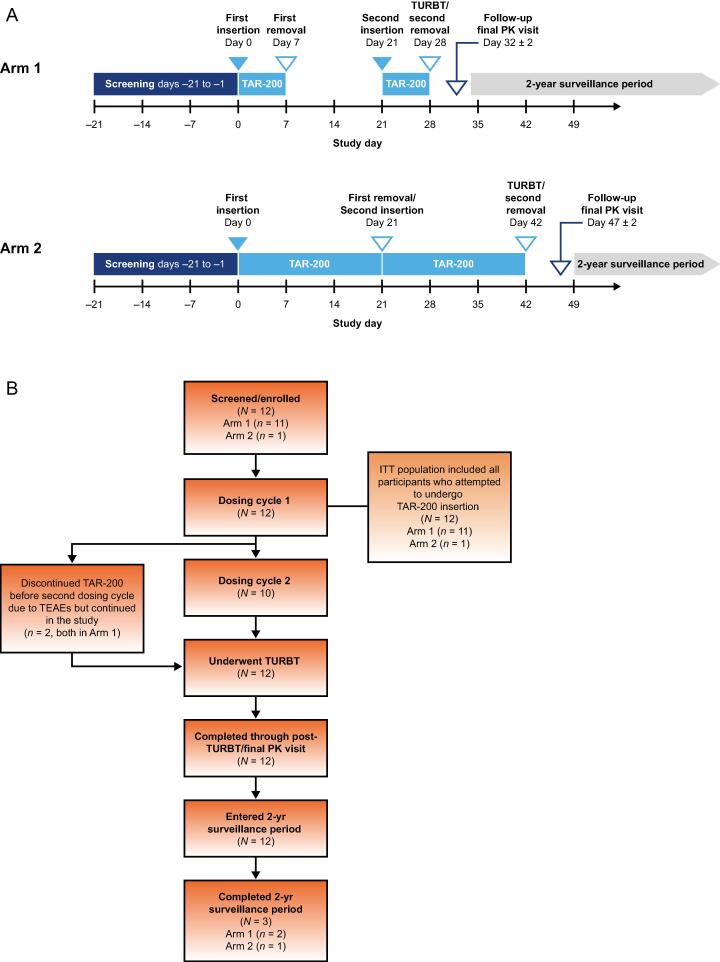
(A) Schema of TAR-200-102 study design. (B) Participant disposition. ITT = intent to treat; PK = pharmacokinetics; TEAE = treatment-emergent adverse event; TURBT = transurethral resection of the bladder tumour.

TAR-200-102 was a prospective, open-label, phase 1b study from July 27, 2016 to March 3, 2020, in which eligible participants with IR NMIBC from two study sites in the Netherlands received TAR-200 in either two 7-d or two 21-d dosing cycles over a 4–6-wk period in a marker lesion/ablation design, between diagnostic cystoscopy and complete TURBT (Fig. 2A). In arm 1, participants received two 7-d TAR-200 dosing cycles, with the initial TAR-200 inserted on day 0 and removed on day 7. After a 14-d rest period, a second TAR-200 was inserted on day 21. Subsequent TURBT or bladder biopsy was performed on day 28. Arm 1 completed enrolment prior to start of arm 2. In arm 2, TAR-200 was dosed in two consecutive 21-d cycles, with the insertion of the first TAR-200 on day 0 and the replacement on day 21. TURBT or bladder biopsy was performed on day 42. A post-TURBT/final PK visit (days 32 and 47 for arms 1 and 2, respectively) occurred before participants entered a 2-yr surveillance period to assess for recurrence. Arm 2 was terminated early for nonclinical reasons at the sponsor’s discretion. Study enrolment was not discontinued for safety concerns.

This study (NCT02720367) was conducted in accordance with the Declaration of Helsinki; the study protocol was approved by an independent ethics committee.

### 2.2 Patient selection

Signed written informed consent was obtained for each participant. Eligible participants had documented a history of histologically confirmed IR papillary urothelial carcinoma as defined by AUA risk stratification [5]. HR NMIBC including carcinoma in situ (CIS) or clinical stage cT1 was excluded. The exclusion criteria included intravesical therapy within 1 yr prior to enrolment, active treatment for other neoplastic disease ≤ 3 mo before enrolment (except hormonal therapy for prostate cancer), pelvic radiotherapy < 6 mo prior to enrolment, evidence of current bladder perforation, active urinary tract infection (UTI), gemcitabine hypersensitivity, concomitant immunosuppressive medication, or anatomical features that would prevent the indwelling use, safe placement, or removal of TAR-200.

### 2.3 TAR-200 drug delivery system

TAR-200 has been described previously [14]. The transurethral placement of TAR-200 was performed with a copackaged, sterile, single-use urinary placement catheter (UPC).

### 2.4 Outcomes

The primary endpoint was safety of TAR-200 and UPC throughout the primary study period, defined as the period from the first TAR-200 insertion to post-TURBT/PK visit. Safety was evaluated by adverse events (AEs), clinical laboratory tests, vital signs, physical examinations, investigational product events (IPEs), bladder postvoid residual (PVR) volume, and cystoscopy findings. An AE was described as any disease-related, treatment-related, or unrelated untoward medical event. IPEs included any observation of TAR-200 or UPC not functioning as intended. Treatment-emergent AEs (TEAEs) were defined as worsening of any pre-existing or new AEs that occurred after the first TAR-200 insertion. TEAEs and serious AEs (SAEs) were recorded from documented informed consent through complete TURBT; thereafter, only SAEs were reported. AEs were coded using the Medical Dictionary for Regulatory Activities version 20.1, and their severity were evaluated according to the National Cancer Institute Common Terminology Criteria for Adverse Events version 4.0. AE relatedness to TAR-200 or to the insertion/removal procedure was investigator assigned.

The secondary endpoints included tolerability of TAR-200 and UPC, PK for gemcitabine and dFdU, and preliminary efficacy, as expressed by complete response (CR) and partial response rates. Tolerability was defined as not requiring unscheduled TAR-200 removal due to predefined safety criteria (ie, gross haematuria requiring transfusion, grade ≥ 2 aseptic cystitis, allergic reaction, TAR-200–related urinary retention, severe infection, clinically significant active UTI, and signs of systemic gemcitabine toxicity) or any grade ≥ 3 TAR-200–related TEAEs. PK analyses were assessed by plasma and urine concentrations of gemcitabine and dFdU. Pathological CR rate was defined as the proportion of participants with pathologically confirmed T0 disease in the post-treatment surgical specimen obtained by TURBT.

Pathological clinical response was evaluated on days 28 and 42 for arms 1 and 2, respectively. If residual tumour was present, TURBT was performed. If none was present, a bladder biopsy was taken at the prior tumour site and surrounding tissue.

Assessments during the 2-yr surveillance included cystoscopy, cytology, haematology, blood chemistry, and urine analysis and cultures.

### 2.5 Statistical analysis

As this was a phase 1b study, there were no formal statistical analyses. Safety, tolerability, PK, and preliminary efficacy are summarised descriptively. Categorical variables were summarised using counts and percentages. As only one participant was enrolled in arm 2, results described are for all participants combined (arms 1 + 2). All analyses were performed using SAS statistical software version 9.4 (SAS Institute, Cary, NC, USA) on the intent-to-treat population, which included all participants enrolled for whom the TAR-200 insertion procedure on day 0 was attempted, irrespective of whether performed successfully or not.

## 3 Results

### 3.1 Patient disposition and demographics

Twelve participants were screened and enrolled (11 in arm 1 and one in arm 2; Fig. 2B). All 12 participants received one or more doses of TAR-200. Two participants did not undergo a second dosing cycle due to TEAEs reported during the first dosing cycle; however, they continued the study and underwent TURBT and post-TURBT/final PK visit. Overall, all 12 participants completed the study by concluding the primary study period and subsequently entered the surveillance period. Three participants completed 2 yr of follow-up, and nine ended the study during the surveillance period due to recurrent disease.

Baseline characteristics are presented in Table 1. All patients had papillary disease and were considered to have an IR by AUA risk stratification [5]. By EAU guidelines, all patients had an IR except one patient who was considered to have an HR [4].

**Table 1:** Demographics and baseline characteristics

Participant demographics	TAR-200 (*N* = 12)
Age (yr), median (Q1, Q3)	70 (65, 74)
Sex, *n* (%)	
Male	9 (75)
Female	3 (25)
White*, n* (%)	12 (100.0)
BMI (kg/m^2^), median (Q1, Q3)	29 (26, 31)
Diesel exhaust exposure, *n* (%)	
No exposure	8 (67)
Low exposure	3 (25)
Medium exposure	0
High exposure	1 (8)
Asbestos, *n* (%)	
No exposure	9 (75)
Low exposure	2 (17)
Medium exposure	1 (8)
High exposure	0
Other, *n* (%)	
No exposure	0
Low exposure	1 (8)
Medium exposure	0
High exposure	0
Not reported	11 (92)
Smokers (current and former), *n* (%)	8 (67)
Prior TURBT, *n* (%)	7 (58)
Prior intravesical immunotherapy or chemotherapy, *n* (%) a	5 (42)
Prior pelvic radiation, *n* (%)	1 (8)
Primary tumour staging and grade at diagnosis, *n* (%)	
Ta, low grade	11 (92)
Ta, high grade	1 (8) b

AUA = American Urological Association; BMI = body mass index; NMIBC = non–muscle-invasive bladder cancer; Q = quartile; Ta = noninvasive papillary carcinoma; TURBT = transurethral resection of the bladder tumour.

a At least 1 yr between last intravesical therapy and enrolment to conform with inclusion criteria.

b Participant was considered to have an intermediate risk by AUA NMIBC risk stratification [5].

### 3.2 Safety and tolerability

All 12 participants (100%) underwent uncomplicated, successful first TAR-200 insertion; the second was successful in all ten participants undergoing second placement. Successful insertion was defined as TAR-200 appearing free of damage and in an appropriate configuration following insertion, as evaluated by cystoscopy. Overall, 11 participants (92%) experienced TEAEs, which were all grade ≤ 2. The most common TEAEs are shown in Table 2. TEAEs experienced by the participant in arm 2 were consistent with those in arm 1. TAR-200–related TEAEs occurred in nine participants (75%) and were all grade ≤ 2; the most frequent were congruent with reported unrelated TEAEs (Table 2). Four participants (33%) reported one or more procedure-related (UPC-related and/or cystoscopy-related) TEAEs, which included haematuria (three participants [25%]), penile pain (two participants [17%]), and malaise, UTI, and noninfective cystitis (one participant [8%] each). No TAR-200–related SAEs occurred during the primary study period; one SAE of severe UTI unrelated to TAR-200 occurred during the surveillance period. No on-study deaths were reported. One IPE was reported: TAR-200 was visualised as coiled rather than the expected pretzel-like configuration following the second dosing cycle due to improper removal not in accordance with the instructions for use. Bladder PVR volume changes were not clinically significant. Cystoscopy findings were consistent with observations in this population.

**Table 2:** Summary of frequent TEAEs by preferred term and grade

Participants with events, *n* (%) a	*N* = 12			
	All	Grade 1	Grade 2	Grade ≥3
Any TEAEs	11 (92)	7 (58)	4 (33)	0
Urgency	7 (58)	5 (42)	2 (17)	0
Dysuria	5 (42)	4 (33)	1 (8)	0
Haematuria	5 (42)	5 (42)	0	0
Constipation	4 (33)	4 (33)	0	0
Penile pain	3 (25)	3 (25)	0	0
TAR-200–related TEAEs	9 (75)	5 (42)	4 (33)	0
Urgency	6 (50)	4 (33)	2 (17)	0
Dysuria	4 (33)	2 (17)	2 (17)	0
Haematuria	4 (33)	4 (33)	0	0
Penile pain	2 (17)	2 (17)	0	0

TEAE = treatment-emergent adverse event.

a TEAEs were reported in ≥ 15% of participants.

All participants were considered tolerant of TAR-200, with no early removals due to TAR-200–related TEAEs or because of meeting predefined safety criteria. No participant discontinued the study due to TEAEs; however, two participants (17%) did not undergo the second dosing cycle due to TAR-200–related and procedure-related TEAEs experienced during the first cycle: one participant had TEAEs of mild haematuria, penile pain, and moderate urinary urgency, and one had mild dysuria, urgency, urinary incontinence, and abdominal pain.

Two UPC-related TEAEs occurred: one participant had mild UTI and one reported mild haematuria.

### 3.3 Pharmacokinetics

**Fig. 3:**
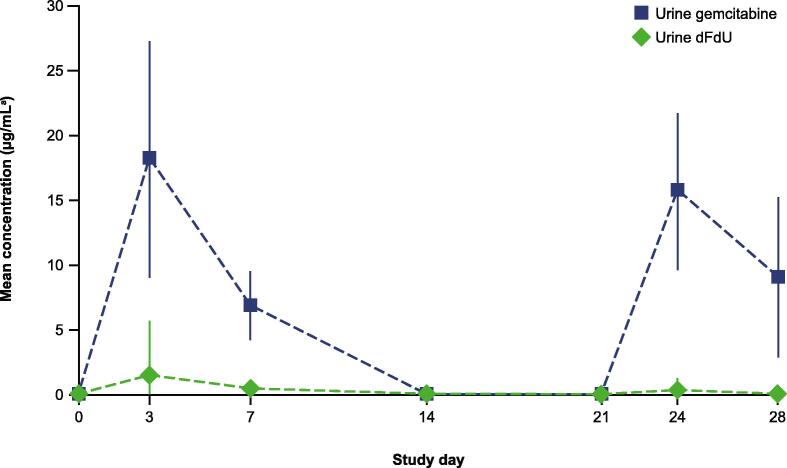
Urine pharmacokinetics during TAR-200 dosing. Vertical bars represent SD. dFdU = 2′,2′-difluorodeoxyuridine; SD = standard deviation. ^a^*N* = 12.

No systemic plasma gemcitabine concentrations were detected in any samples. Plasma concentrations of dFdU were observed infrequently, with very low measurable concentrations that ranged from 0.101 to 0.163 µg/ml found in 3% of samples analysed. Of note, gemcitabine plasma concentrations > 15 µM may result in increased myelosuppression [20]. Urine gemcitabine concentrations were detectable 7 d after instillation (Fig. 3). The maximum mean gemcitabine urine concentrations occurred 3 d following the insertion for both dosing cycles and ranged between 18.2 and 15.7 µg/ml. Urine concentrations of dFdU were generally lower than gemcitabine concentrations at observed time points and ranged between 1.48 and 0.36 µg/ml, suggesting uptake and tissue saturation of this metabolite [21].

### 3.4 Preliminary efficacy

Response at TURBT was assessed for all participants in the intent-to-treat population (*N* = 12). All 12 participants attempted to undergo TURBT; however, one participant had no visible tumour at either location where the tumour was initially observed, so biopsy/pathology was not performed. This participant was considered as having CR based on visual assessment at cystoscopy. Overall, five participants (42%) had CR. The remaining participants had visible residual disease; six had Ta (grade 2a, *n* = 3; grade 1, *n* = 2; unknown grade, *n* = 1) and one had CIS (grade 3). During the surveillance period, cystoscopic examinations detected suspicious areas of disease in ten participants. Nine participants (75%) ended study participation once findings were detected. As per the local standard of care with cystoscopic follow-up of small papillary lesions, four of these participants (classified as three responders and one nonresponder at TURBT) underwent biopsies; three had low-grade Ta and one had high-grade Ta.

## 4 Discussion

The preliminary results of this phase 1b study show that the placement of two sequential TAR-200 intravesical delivery systems was safe and well tolerated in participants with IR NMIBC. The TEAEs observed are anticipated in participants with IR NMIBC due to disease state and prestudy medical history, and are also congruent with urethral catheterisation, cystoscopy, and intravesical instillations [22], [23], [24], [25]. No TEAE-related discontinuations, on-study deaths, or SAEs were reported during the primary study period. Both TAR-200 and the UPC were considered safe with no unanticipated safety findings attributed to TAR-200 or UPC.

Prolonged exposure to an intravesical chemotherapeutic drug has previously been shown to have an increased cytotoxic effect on bladder tumour cells [26], [27]. Other methods that prolong tumour exposure to drugs, such as hydrogels, liposome-gel complexes, and conventional approaches to reduce frequent urination, have been evaluated [28], [29], [30]. While these methods may increase exposure for up to several hours, TAR-200 enables drug exposure for at least 7 d. The prolonged exposure elicited by TAR-200 is corroborated by urine PK data demonstrating peak urine concentrations of gemcitabine at day 3, with the mean concentration of gemcitabine detectable in urine at > 5 μg/ml up to 7 d after insertion (Fig. 3). Importantly, no measurable gemcitabine levels were detected in systemic circulation.

While recent studies have proposed the use of active surveillance and in-office fulguration for the management of low-grade tumours, further studies to determine the long-term effectiveness of these treatment options are needed [31], [32]. Furthermore, TAR-200 insertion and removal are considered minimally invasive and may be well suited for the recent trend of deintensification of treatments for low-grade tumours sometimes observed in IR NMIBC.

Placement of two TAR-200 drug delivery systems showed preliminary efficacy with a CR rate of 42%, including pathological CR observed in four participants and CR based on visual assessment at cystoscopy in one participant. It is noteworthy that two of seven participants with no response had completed only one of two 7-d dosing cycles, so it is possible that additional induction or maintenance cycles may have improved response rates. The CR rate via tumour ablation observed with TAR-200 compares favourably with other marker lesion/ablation studies utilising gemcitabine and suggests potential merit in the premise of sustained low-dose drug delivery compared with standard intravesical therapy [33], [34], [35], [36].

The early cessation of arm 2 did not allow for a comparator to assess optimal dosing. Given its phase 1 design, this study is limited by its small sample size; single-arm, induction therapy–only design; and difficulty with the assessment of partial response in participants with multifocal tumours. Additional limitations included the challenge to determine whether TAR-200–related TEAEs were due to the device or the drug component as these are integral. As this was a phase 1b study with a relatively short follow-up period, antitumour activity of TAR-200 was noted, but there was difficulty in evaluating the durability of the observed CRs, and not all patients with suspected recurrences during the surveillance period underwent biopsies.

Given the promising ablation noted after two relatively brief indwelling periods, the results from this study further support the safety and potential efficacy of TAR-200 in IR NMIBC, while data from the TAR-200-101 (NCT02722538) [14] and TAR-200-103 (NCT03404791) [16] studies demonstrated the beneficial preliminary efficacy of TAR-200 in MIBC. To further investigate the safety and efficacy of TAR-200 as monotherapy and in combination with an immune checkpoint inhibitor, large-scale, randomised, controlled, global studies are underway in the SunRISe clinical trial program (NCT04640623, NCT04658862, NCT04919512, and NCT05714202). Recently presented preliminary data from SunRISe-1 in an HR NMIBC bacillus Calmette–Guérin-unresponsive population further support the efficacy of TAR-200, with a CR rate of 77% (95% confidence interval, 58–90) in the TAR-200–alone arm (*n* = 30). At a median follow-up of 48 wk, the median duration of response was not reached, demonstrating good durability [37]. These and future studies will provide important clarity on both the benefits and the risks of sustained local drug delivery in the urinary tract, and insights into how these innovative treatments may potentially transform care for bladder cancer patients.

## 5 Conclusions

In this phase 1b study, TAR-200 appears to be safe and well tolerated in participants with IR NMIBC. Preliminary, intravesical TAR-200 monotherapy showed beneficial antitumour effects via direct papillary tumour ablation, with a CR rate of 42% after two consecutive TAR-200 placements prior to TURBT. Further evaluation of safety, biomarker, quality of life, and efficacy data in HR NMIBC and MIBC is ongoing in multiple phase 2 and 3 global studies.

  ***Author contributions*:** F. Johannes P. van valenberg had full access to all the data in the study and takes responsibility for the integrity of the data and the accuracy of the data analysis.

  *Study concept and design*: Cutie, Maffeo, van Valenberg, Witjes.

*Acquisition of data*: All authors.

*Analysis and interpretation of data*: All authors.

*Drafting of the manuscript*: All authors.

*Critical revision of the manuscript for important intellectual content*: All authors.

*Statistical analysis*: Stromberg, Li.

*Obtaining funding*: Cutie.

*Administrative, technical, or material support*: Bhanvadia, Keegan, Sweiti.

*Supervision*: Bhanvadia, Keegan, Sweiti.

*Other*: None.

  ***Financial disclosures***: F. Johannes P. van valenberg certifies that all conflicts of interest, including specific financial interests and relationships and affiliations relevant to the subject matter or materials discussed in the manuscript (eg, employment/affiliation, grants or funding, consultancies, honoraria, stock ownership or options, expert testimony, royalties, or patents filed, received, or pending), are the following: F.J.P. van Valenberg, A.G. van der Heijden, J.P.M. Sedelaar, J.J.O. Steenbruggen, D.M. Somford, and J.A. Witjes have no financial or other conflict of interest related to this study. C.J. Cutie, C. Iarossi, A. Kelley, and J.C. Maffeo are former employees of TARIS Biomedical and are employees of Janssen Pharmaceuticals. S. Bhanvadia, K.A. Keegan, S. Hampras, H. Sweiti, S. Jin, X. Li, and K.A. Stromberg are employees of Janssen Pharmaceuticals. A. Chau and D.L. Reynolds were paid consultants for TARIS Biomedical and Janssen Pharmaceuticals.

  ***Funding/Support and role of the sponsor:*** This study was sponsored by TARIS Biomedical, now part of Janssen.

  **:*Data sharing*** The data sharing policy of Janssen Pharmaceutical Companies of Johnson & Johnson is available at https://www.janssen.com/clinical-trials/transparency. As noted on this site, requests for access to the study data can be submitted through the Yale Open Data Access (YODA) Project site at http://yoda.yale.edu.

  ***Acknowledgments***: We would like to thank Sandra Mengede, Marion van der Putten, Elze Verbeek-Camps, Petra Frenken, Margot Polfliet, and Wendy Zegers for their supportive role in the completion of this study. Writing assistance was provided by Nicolisha Narainpersad, PhD, of Parexel, and was funded by Janssen Global Services, LLC. This manuscript reports data on the investigational use of a product or device not yet approved by the FDA for any purpose. This study was previously presented at ASCO Genitourinary Cancers Symposium, February 16–18, 2023.
